# Overcoming Hole‐Extraction Barriers: A Facile PEDOT:PSS Interlayer Unlocks Record‐Low Voltage Deficit in PbS Quantum Dot Solar Cells

**DOI:** 10.1002/advs.202524275

**Published:** 2026-01-09

**Authors:** Shengkai Kang, Zixuan Meng, Yabing Wang, Kanwen Zheng, Sibo Huang, Di Zhang, Shitong Li, Cong Chen, Shenqing Ren, Yang Li, Chao Ding, Dewei Zhao

**Affiliations:** ^1^ College of Materials Science and Engineering & Institute of New Energy and Low‐Carbon Technology Engineering Research Center of Alternative Energy Materials & Devices of Ministry of Education Sichuan University Chengdu China

**Keywords:** interface engineering, PbS quantum dot solar cells, PEDOT:PSS, voltage deficit

## Abstract

The development of colloidal quantum dot solar cells (CQDSCs) is currently constrained by the substantial open‐circuit voltage (*V*_oc_) deficit and intricate fabrication processes. Here, we present a simplified device architecture achieved by synergistically combining a direct‐synthesis colloidal quantum dot (CQD) ink with a facile methanol/oxalic acid modification of the Poly(3,4‐ethylenedoxythiophene):polystyrene sulfonate (PEDOT:PSS) interlayer. This strategy simultaneously addresses the poor wettability of aqueous PEDOT:PSS on hydrophobic PbS‐EDT layers and initiates critical chemical optimization. By selectively removing insulating PSS, the treatment fosters a dense, electronically uniform fibrous network. Crucially, this optimized interlayer exhibits a work function shift from −5.30 to −5.15 eV, which reduces the hole extraction barrier to the Ag electrode from 0.51 to 0.36 eV. This favorable energy alignment extends the carrier lifetime from 0.54 to 2.37 ms and accelerates charge extraction. Consequently, the *V*_oc_ is boosted from 649 to 742 mV, propelling the power conversion efficiency to 13.97%. This work offers a robust, process‐compatible interfacial strategy to unlock stable, high‐voltage CQDSCs.

## Graphical abstract

A synergistic methanol and oxalic acid interface engineering strategy is developed for PbS quantum dot solar cells. This approach transforms the PEDOT:PSS layer into a dense, electronically uniform, and conductive network. Consequently, the device achieves a record‐low voltage deficit of 340 mV, yielding a remarkable *V*_oc_ of 742 mV and a power conversion efficiency of 13.97%.

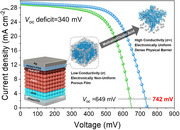

## 1 Introduction

Colloidal quantum dots (CQDs), particularly those based on lead sulfide (PbS), have emerged as stellar candidates for next‐generation photovoltaics. Their unique size‐tunable bandgap in the near‐infrared (NIR) spectrum makes them an ideal partner for wide‐bandgap perovskites in all‐solution‐processed tandem solar cells, poised to surpass the single‐junction efficiency limit [1, 2, 3]. However, the viability of this tandem architecture hinges critically on the performance of the bottom cell, specifically, its ability to generate a high open‐circuit voltage (*V*_oc_) from low‐energy photons. To date, a substantial *V*_oc_ deficit (*V*_oc_ loss = *E*_g_/*q*‐*V*_oc_), stemming from severe non‐radiative recombination at interfaces, has remained the primary bottleneck, not only limiting single‐junction performance but also hindering the progress of high‐efficiency tandem devices [4, 5].

A dual challenge comes within the field. On one hand, mitigating the critical voltage loss has necessitated increasingly intricate interface engineering, including multi‐junction passivation and the development of novel, complex hole transport materials (HTLs) [6, 7]. On the other hand, to streamline device fabrication, room‐temperature direct synthesis of semiconducting CQD inks has recently emerged as a transformative approach [8, 9]. This strategy enables the deposition of high‐quality active layers in a single step, dramatically reducing cost and enhancing scalability [10]. A contradiction thus arises: the complex interface modifications required for high performance often negate the process simplicity offered by advanced ink formulations.

This dilemma is further compounded by the selection of electrode materials. To date, most reported high‐performance PbS quantum dot solar cells (QDSCs) with power conversion efficiencies (PCEs) exceeding 13% have relied on expensive gold (Au) electrodes to ensure efficient hole collection [6, 8, 11]. Replacing such noble metals with more abundant alternatives, such as silver (Ag), is a critical step for broader applicability. However, we found that direct deposition of Ag onto the primary PbS‐EDT hole transport layer (HTL) results in poor device performance and frequent short‐circuiting. Poly(3,4‐ethylenedoxythiophene):polystyrene sulfonate (PEDOT:PSS) is an ideal candidate as a functional interlayer; it is a mature, solution‐processable material whose chemical, physical, and electronic properties are highly tunable via doping or solvent‐based treatments [12, 13].

Our prior work has established that the HTL‐side interfaces are the dominant factor limiting performance, and theoretical simulations have compellingly pinpointed the final HTL/back contact interface—which, based on the above, we identify as the PbS‐EDT/PEDOT:PSS/Ag junction—as the next critical frontier for breaking performance barriers [11, 14]. Various acid treatments have been explored to tune the work function and conductivity of PEDOT:PSS, typically by promoting phase separation and removing insulating PSS [15, 16, 17, 18]. However, the specific mechanism by which oxalic acid (OA) modifies this electronic junction has not been fully explored. Critically, it remains to be seen whether this strategy can resolve the dilemma between device performance and process simplicity.

Herein, we report a facile strategy for tailoring the critical hole‐extraction interface via a synergistic methanol (MeOH) and OA modification. We fabricated devices using a scalable direct‐synthesis PbS CQD ink while introduced a facile single‐step synergistic modification (MeOH + OA) at the theoretically critical hole‐extraction interface. This approach is particularly critical, as it simultaneously addresses two key challenges: (i) the physical processing barrier associated with depositing aqueous PEDOT:PSS onto the hydrophobic PbS‐EDT layer, and (ii) the systematic optimization of interfacial chemical, physical, and electronic properties relevant to device performance. Our strategy unlocks a record‐high *V*_oc_ of 742 mV and boosts the PCE to 13.97%. Ultimately, this work establishes a mechanism‐guided, process‐compatible interfacial strategy as a robust blueprint for scalable, high‐efficiency quantum dot photovoltaics.

## 2 Results and Discussion

**FIGURE 1:**
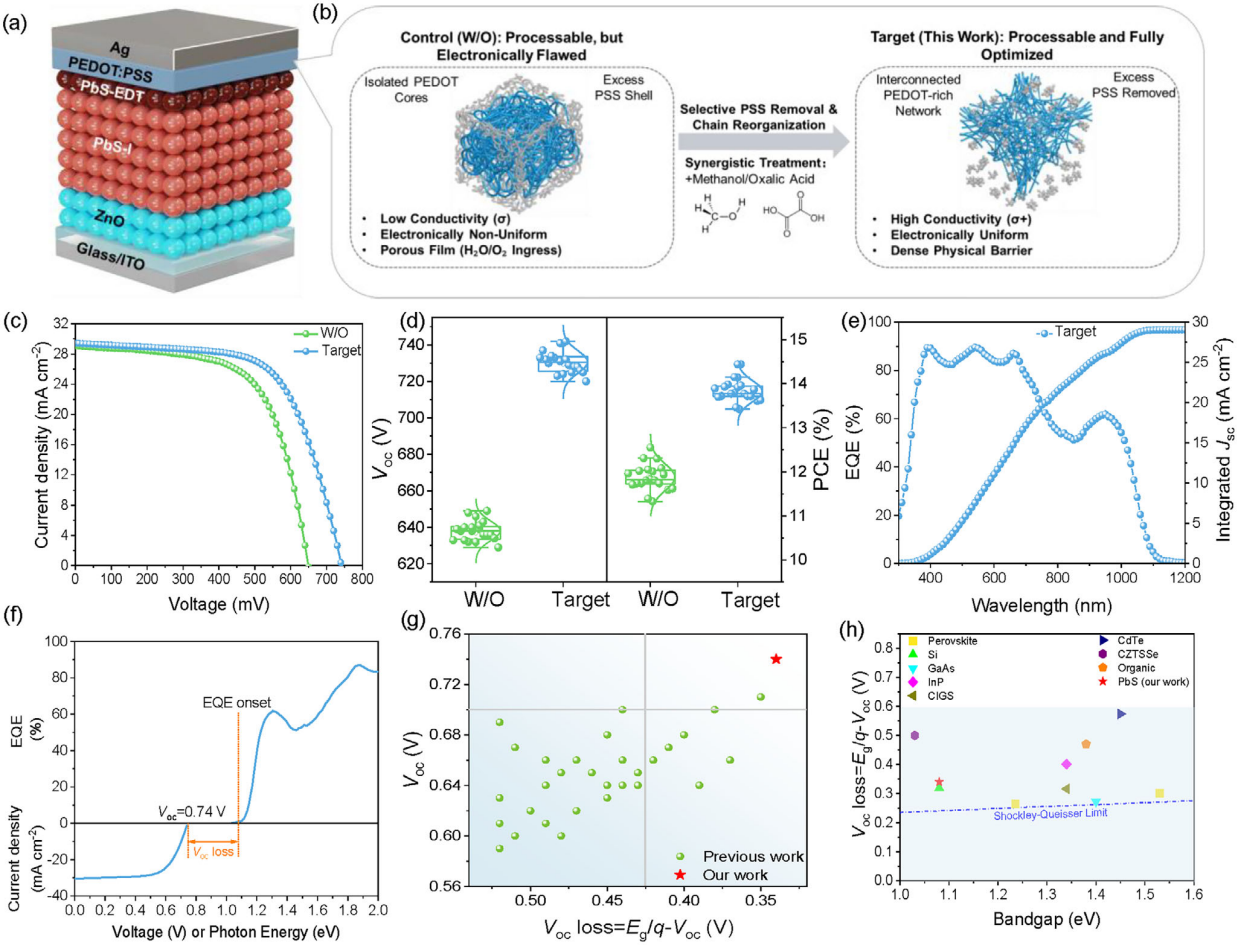
a) Device structure of the solar cell. b) Schematic comparison of the hole‐extraction interface formation. c) *J–V* characteristics of the champion control (W/O) and target devices under simulated AM 1.5G illumination. d) Statistical distribution of *V*_oc_ and PCE obtained from 20 independent devices for each group (*n* = 20). e) EQE spectrum and the corresponding integrated *J*_sc_ for the champion target device. f) EQE spectrum (top) and *J–V* curve (bottom) for the record‐efficiency device. g) Benchmarking of versus *E*_g_ for high‐efficiency (PCE > 10%) PbS QDSCs. h) Comparison of the *V*_oc_ loss for this work against other state‐of‐the‐art photovoltaic technologies. Note: The label “W/O” denotes the control sample treated with Methanol only (without Oxalic Acid), distinguishing it from the pristine (untreated) PEDOT:PSS.

We constructed the device architecture of ITO/ZnO/PbS‐PbI_2_/PbS‐EDT/PEDOT:PSS/Ag (Figure 1a; Figure S1), with the distinct layer stacking confirmed by the cross‐sectional SEM image in Figure S1b. However, depositing aqueous PEDOT:PSS onto the hydrophobic PbS‐EDT layer presents severe processing challenges. As shown in Figure S2, pristine PEDOT:PSS exhibits poor wettability (contact angle ∼43.6°), while direct addition of OA causes solution gelling and further degrades wettability (∼69.6°), rendering it unprocessable. To overcome this, we developed a synergistic (MeOH + OA) treatment. MeOH acts as a critical wetting agent and rheological modifier, enabling uniform film formation. We thus defined the MeOH‐treated film as Control (W/O) and the MeOH/OA‐treated film as Target.

Figure 1b illustrates the proposed transformation mechanism. The Control interface exhibits a granular, PSS‐rich morphology. This suboptimal structure leads to low conductivity, electronic inhomogeneity, and poor stability. Critically, we also identify a severe work function misalignment at this interface as the key bottleneck creating a hole extraction barrier.

Conversely, the Target interface (right) utilizes OA as a chemical engine to selectively remove excess PSS and trigger chain reorganization. We posit that this chemical modification creates a dense, interconnected PEDOT‐rich network that simultaneously enhances conductivity, electronic uniformity, and physical stability. Furthermore, this reorganization is hypothesized to precisely tune the interlayer's work function, creating an optimized “energy staircase” that drastically lowers the final extraction barrier. In the following sections, we provide comprehensive experimental evidence to validate this multi‐faceted hypothesis.

The profound impact of our interfacial engineering is evident in photovoltaic performance (Figure 1 and Table 1). The Current density‐voltage (*J–V*) curves of the champion devices (Figure 1c) reveal a dramatic performance leap. As detailed in Table 1, the PCE improves from 12.01% for the control device to 13.97% for the Target device. This performance boost is driven almost exclusively by a substantial rise in *V*_oc_ from 649 to 742 mV, while the *J*_sc_ and FF remain negligible changes.

**TABLE 1:** Summary of photovoltaic parameters for PbS QDSCs. The values outside parentheses represent the champion device performance. The values in parentheses indicate the mean ± standard deviation derived from 20 independent devices for each group (*n* = 20).

Sample	*V*_oc_ (mV)	*J*_sc_ (mA/cm^2^)	FF (%)	Champion PCE (average PCE) (%)
W/O	649 (638.1 ± 5.4)	29.08 (29.84 ± 0.60)	63.6 (62.4 ± 1.0)	12.01 (11.88 ± 0.30)
Target	742 (730.7 ± 6.2)	29.42 (29.99 ± 0.52)	64.1 (63.1 ± 1)	13.97 (13.83 ± 0.24)

This performance enhancement is highly reproducible, as confirmed by the statistical analysis of 20 devices (Figure 1d). The target devices exhibit a consistently higher average *V*_oc_ of 730.7 ± 6.2 mV and an average PCE of 13.83 ± 0.24%, with similar trends in *J*_sc_ and FF (Figure S3). The external quantum efficiency (EQE)‐integrated current density is 28.78 mA/cm^2^ (Figure 1c), which exhibits a negligible mismatch of ∼2.2% with the *J_sc_* obtained from the *J–V* measurements (29.42 mA/cm^2^), confirming its consistency [19]. Meanwhile, the corresponding EQE spectrum of the control device (Figure S4) yields a similar integrated *J*_sc_ of 28.21 mA/cm^2^.

To contextualize the significance of such high *V*_oc_, we analyzed the voltage deficit using the EQE onset (Figure 1f), determined to be ∼1.08 eV [4, 20]. This, combined with our champion *V*_oc_ of 742 mV, yields a remarkably low *V*_oc_ deficit of only 340 mV. Benchmarking against state‐of‐the‐art PbS QDSCs (Figure 1g; Table S1) positions our device at the upper frontier. Furthermore, this reduction significantly advances PbS QDSCs among leading PV technologies (Figure 1h; Table S2). This data unequivocally establishes our strategy's effectiveness in achieving record‐level *V*_oc_, the physical mechanisms of which are detailed in subsequent sections.

**FIGURE 2:**
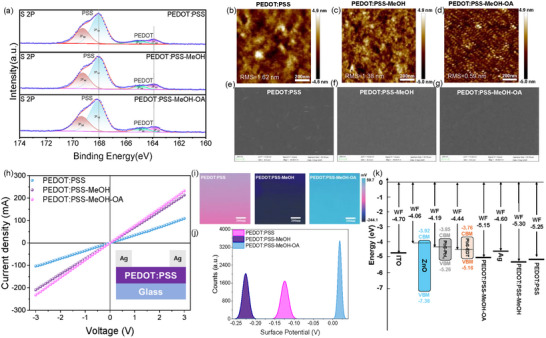
Comprehensive chemical, morphological, and electronic characterizations of the PEDOT:PSS interlayers. a) High‐resolution S 2p XPS spectra for the pristine PEDOT:PSS, PEDOT:PSS‐MeOH (W/O device), and PEDOT:PSS‐MeOH‐OA (Target device) films. b–d) AFM topography images and e–g) corresponding SEM images for the (b,e) pristine, (c, f) PEDOT:PSS‐MeOH, and (d, g) PEDOT:PSS‐MeOH‐OA films. h) *I–V* characteristics of the three films, measured using the Ag/PEDOT:PSS/Glass structure (inset), to evaluate film conductivity. i) KPFM surface potential maps and (j) their corresponding statistical distribution histograms for the three film types. k) Energy level diagram of the device stack, constructed from UPS and optical absorption data (detailed in Figures S8 and S9), illustrating the energy level alignment for the pristine, W/O, and Target conditions.

To understand the origin of the performance enhancement, we systematically investigated the chemical, morphological, and electronic properties of the PEDOT:PSS interlayers (Figure 2). First, high‐resolution x‐ray photoelectron spectroscopy (XPS) of the S 2p core level (Figure 2a) reveals the chemical origin [21, 22]. Quantitative analysis (Table S3) shows a dramatic increase in the PEDOT:PSS atomic ratio for the synergistic (MeOH + OA) treatment, providing direct evidence of selective insulating PSS removal [23, 24]. This chemical transformation is further corroborated by Fourier transform infrared spectroscopy (FTIR) analysis (Figure S5), where a marked decrease in the relative intensity of PSS‐related vibrations (e.g., S═O stretching) is observed for the target film [25, 26]. Furthermore, pH measurements (Figure S6) decouple this enhancement from simple de‐acidification, as the optimal Target solution is the most acidic (pH = 1.76), confirming that the improvements stem from specific chemical interactions of OA [25, 27, 28]. While simple pH adjustments alone cannot account for the full enhancement, the specific chemical nature of OA plays a pivotal role. We selected OA primarily because it possesses a significantly higher acidity (*pK*_a1_≈1.25) compared to common weak organic acids (e.g., acetic acid, *pK*_a_≈4.76). This specific acidity enables effective proton transfer to the PSS sulfonate anions, disrupting the ionic PEDOT:PSS crosslinks [29]. Furthermore, the small molecular size and dicarboxylic structure of OA allow for efficient diffusion into the polymer matrix, facilitating the removal of insulating PSS chains that weaker or bulkier acids cannot achieve [30].

This fundamental chemical transformation directly drives the dramatic morphological evolution observed via atomic force microscopy (AFM) and scanning electron microscopy (SEM) (Figure 2b–g). While the pristine and W/O films (Figure 2b,c,e,f) show a relatively rough (RMS = 1.62 and 1.38 nm, respectively) and granular morphology, the synergistic treatment (Target) results in a complete metamorphosis. The AFM image (Figure 2d) reveals a highly uniform, ultra‐smooth surface (RMS = 0.59 nm), and the corresponding SEM image (Figure 2g) shows a dense, featureless film. This superior morphology is a direct consequence of the PSS removal, allowing the conductive PEDOT chains to reorganize into an efficient, long‐range transport network [31, 32, 33]. It is important to note that while the acidity of OA aids in phase separation, the enhancement is primarily driven by the structural removal of insulating PSS and the resulting conformational ordering (coil‐to‐linear transition), rather than additional protonation or doping of the PEDOT backbone [25, 30].

Electrically, this optimized morphology leads to improved conductivity. A quantitative analysis of the *I*–*V* slopes (Figure 2h) reveals a reduction in resistance from ∼14.3 Ω (PEDOT:PSS‐MeOH) to ∼13.0 Ω (PEDOT:PSS‐MeOH‐OA). This improvement is further corroborated by Hall effect measurements (Table S4), which show a distinct decrease in resistivity from 1.28 × 10^2^ to 1.14 × 10^2^ Ω·cm. This critical finding indicates that bulk conductivity enhancement is not the primary driver of the performance leap. UV–vis spectroscopy (Figure S7) supports this, showing only a slight increase in near‐infrared absorption consistent with the modest carrier concentration increase, while confirming that optical transparency is maintained. Consequently, the performance improvement must originate from optimized electronic homogeneity and energy‐level alignment [34].

Crucially, kelvin probe force microscopy (KPFM) mapping (Figure 2i,j) reveals a profound improvement in electronic homogeneity. As summarized in Table 2, the potential distribution's FWHM systematically narrows from 22.70 mV (Pristine) and 19.63 mV (W/O) to a remarkably sharp 11.38 mV for the Target film. This signifies the creation of a far more electronically uniform surface, paramount for minimizing local non‐radiative recombination “hotspots” and explaining the substantial *V*_oc_ enhancement.

**TABLE 2:** Summary on key parameters from KPFM analysis. The peak surface potential, peak intensity, and FWHM for the pristine, methanol‐treated (W/O), and target (MeOH + OA) PEDOT:PSS films, extracted from the statistical distribution histograms shown in Figure 2h.

Sample	Potential (mV)	High intensity	FWHM (mV)
PEDOT:PSS	−124.5	1691	22.70
PEDOT:PSS‐MeOH	−225.6	2027	19.63
PEDOT:PSS‐MeOH‐OA	18.3	3491	11.38

Finally, these chemical, physical, and electronic optimizations are integrated into the full device energy level diagram shown in Figure 2k, constructed from detailed ultraviolet photoelectron spectroscopy (UPS) and optical absorption data (Figures S8 and S9). The diagram illustrates the critical role of our interfacial engineering, aligning with recent trends in optimizing PEDOT:PSS for high‐performance optoelectronics [35]. In the W/O device (PEDOT:PSS‐MeOH), a massive 0.51 eV hole extraction barrier exists between the HTL's work function (−5.30 eV) and the Ag electrode's work function (−4.79 eV). This barrier severely impedes hole collection and represents a major performance bottleneck, consistent with theoretical predictions highlighting the importance of the HTL/back contact interface. Our synergistic treatment (PEDOT:PSS‐MeOH‐OA) precisely raises (shallows) the HTL's work function to −5.15 eV, thereby significantly reducing this critical barrier to 0.36 eV [34, 36]. This optimized “energy‐staircase” structure, while still presenting a small barrier, represents a vast improvement. This finding clarifies that the primary extraction barrier is not at the PbS‐EDT/HTL interface. Instead, the limiting bottleneck resides at the HTL/Ag electrode junction. This successfully addresses the key bottleneck predicted by simulations [14]. The combined evidence from Figure 2 thus provides a comprehensive physical and chemical basis for the record‐breaking performance observed in Figure 1.

**FIGURE 3:**
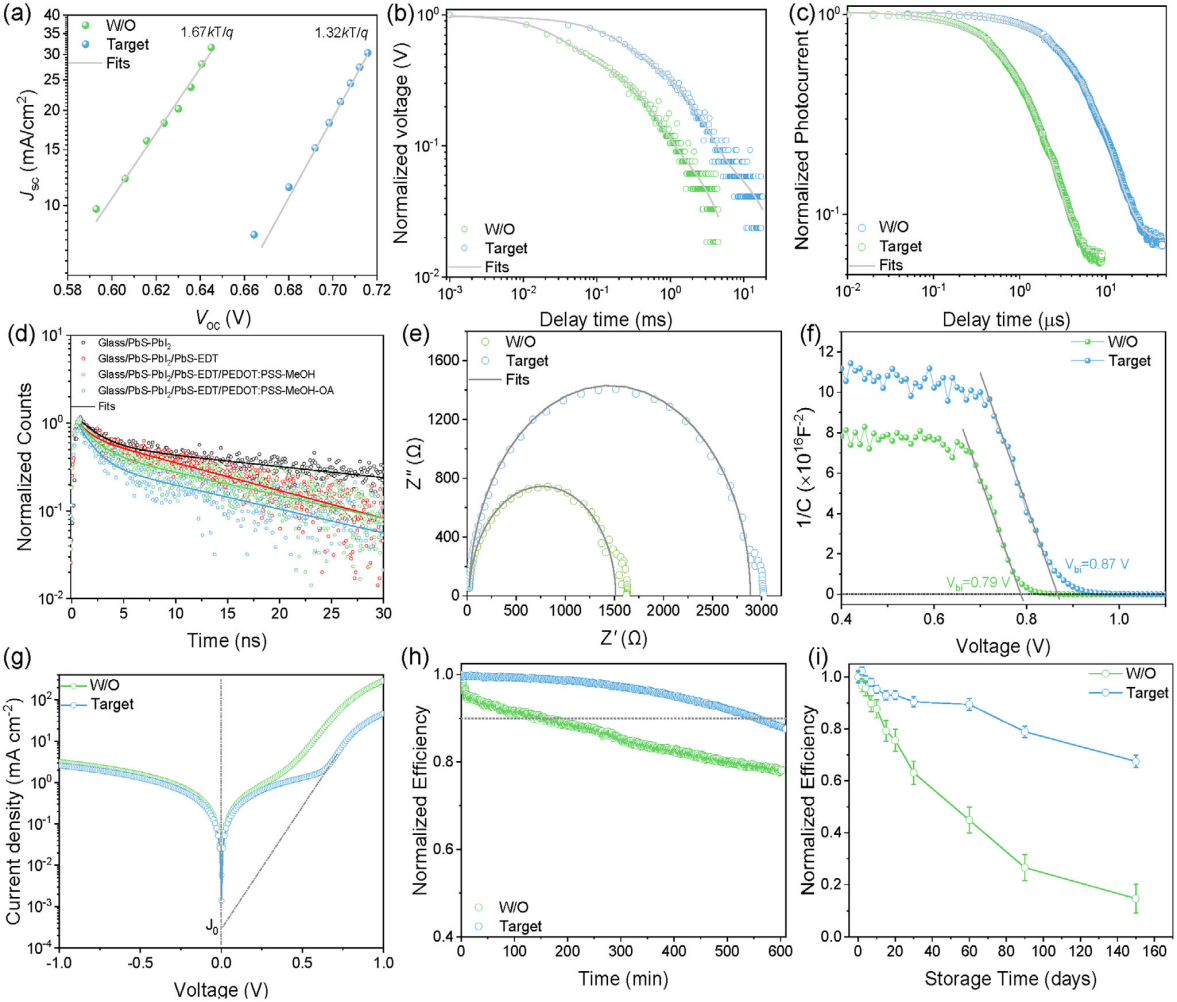
Device physics and carrier dynamics analysis of W/O and Target PbS QDSCs. a) *J*_sc_ as a function of *V*_oc_ under varying light intensities for the W/O and Target devices. b) Normalized TPV decay curves for both devices. c) Normalized TPC decay curves for both devices. d) Normalized TRPL decay curves of PbS‐PbI_2_ films with different HTL configurations: Glass/PbS‐PbI_2_ (black), Glass/PbS‐PbI_2_/PbS‐EDT (red), Glass/PbS‐PbI_2_/PbS‐EDT/PEDOT:PSS‐MeOH (green, W/O), and Glass/PbS‐PbI_2_/PbS‐EDT/PEDOT:PSS‐MeOH‐OA (blue, Target). The solid lines represent bi‐exponential fits. e) EIS Nyquist plots measured in the dark at 0 V bias for both devices. f) Mott‐Schottky plots (1/C^2^ versus V) measured in the dark for both devices, with linear fits to determine the built‐in potential. g) Dark *J–V* characteristics of both devices plotted on a semi‐logarithmic scale. h) Operational stability of the W/O and Target devices, tracked at the MPPT under continuous AM 1.5G illumination. i) Storage stability of unencapsulated W/O and Target devices in a dry air environment.

To quantitatively confirm the superior device operation, we systematically investigated the carrier dynamics (Figure 3). First, the dominant recombination mechanisms were analyzed via light‐intensity‐dependent *V*_oc_ (Figure 3a). The control (W/O) device exhibits a high ideality factor (*n* = 1.67), indicating dominant trap‐assisted (SRH) recombination, consistent with the non‐uniform electronic surface (Figure 2j) and large extraction barrier (Figure 2k) [37, 38]. In contrast, the Target device shows a much‐improved *n* = 1.32, evidencing effective suppression of non‐radiative recombination. This is corroborated by transient photovoltage (TPV) measurements (Figure 3b), where the carrier lifetime increases by 4.4‐fold (from 0.539 to 2.37 ms) in the Target device [39, 40, 41]. This dramatic reduction of recombination is the primary driver for the record‐breaking *V*_oc_.

Charge extraction dynamics were probed using transient photocurrent (TPC) decay measurements (Figure 3c). The W/O device shows a sluggish extraction time (*τ*_ext_ = 6.63 µs) due to low conductivity and the large barrier. In sharp contrast, the target device exhibits an extraction time of only *τ*_ext_ = 1.28 µs, representing a 5.2‐fold acceleration in charge extraction. This rapid extraction is consistent with the optimized interface but, interestingly, did not lead to a massive FF increase, suggesting FF is limited by other factors [42, 43]. To independently validate these kinetics, we performed time‐resolved photoluminescence (TRPL) measurements (Figure 3d). The Target stack exhibits the shortest lifetime (12.97 ns) compared to the W/O stack (14.2 ns), confirming the most efficient hole extraction, which aligns perfectly with the TPC results and strong PL quenching in Figure S10.

We further deconstructed internal losses using electrochemical impedance spectroscopy (EIS) (Figure 3e). The resulting Nyquist plots were fitted using the equivalent circuit model shown in Figure S11. First, the series resistance (*R*_s_), which represents bulk and contact ohmic losses, is reduced from 13.5 Ω·cm^2^ in the W/O device to 9 Ω·cm^2^ in the target device. This modest reduction in *R*_s_ is consistent with the 1.14‐fold conductivity increase (Table S4) and the stable FF. Second, we observe a clear decrease in the charge transfer resistance (*R*_ct_) from 36 Ω·cm^2^ down to 27 Ω·cm^2^. *R*_ct_ quantifies the resistance to hole extraction at the HTL/electrode interface [44, 45]. Most importantly, the recombination resistance (*R*_rec_) shows a substantial increase, from 99 Ω·cm^2^ in the W/O device to 243 Ω·cm^2^ in the target device. This 2.45‐fold increase in *R*_rec_ signifies a greatly suppressed non‐radiative recombination rate, which quantitatively corroborates the 4.4‐fold longer carrier lifetime from TPV (Figure 3b) and the reduced ideality factor (Figure 3a), solidifying this as the key driver for the enhanced *V*_oc_ [46]. This is supported by Mott‐Schottky analysis (Figure 3f), showing a larger built‐in potential (*V*_bi_) of 0.87 V for the Target device (vs 0.79 V for W/O), providing a stronger driving force for charge collection [47]. Finally, dark *J*–*V* characteristics (Figure 3g) show an ∼8‐fold reduction in reverse saturation current (*J*_0_) for the Target device, quantitatively explaining the *V*_oc_ enhancement [48].

We rigorously evaluated device stability. Operational stability (Figure 3h) under continuous AM 1.5G illumination (maximum power point) shows the Target device retaining ∼92.5% of its initial PCE after 500 min, whereas the W/O device degrades to ∼79%. Storage stability in dry air (Figure 3i) further highlights this resilience: while the W/O device drops to ∼15% after 150 days, the Target device retains ∼67.5%. We attribute this enhancement to the dense, ultra‐smooth morphology of the PEDOT:PSS‐MeOH‐OA layer, which acts as a physical barrier blocking oxygen/moisture ingress and suppressing Ag^+^ migration, as hypothesized in our mechanism. Furthermore, the exceptional device performance confirms the thermal stability of the modified interlayer. The treated films withstood the thermal annealing process (70 °C) and the significant radiative heat during Ag electrode evaporation without deterioration, evidencing that organic acids are resistant again the heat processing in our case. This thermal resilience is likely attributed to the removal of labile PSS components and the formation of a densely packed, ordered PEDOT network.

## 3 Conclusion

We have developed a facile synergistic strategy to modify the PEDOT:PSS interlayer using MeOH and OA. This method effectively overcomes the poor processability on hydrophobic PbS‐EDT substrates. Simultaneously, it resolves severe electronic losses at the hole‐extraction interface. By selectively removing insulating PSS, the treatment creates a dense and electronically uniform network. Crucially, it tunes the work function to minimize the energy barrier at the PEDOT:PSS/Ag interface. This optimization effectively suppresses non‐radiative recombination. As a result, we achieve a record *V*_oc_ of 742 mV, a champion PCE of 13.97%, and exceptional operational stability. This work provides a scalable route to efficient and stable devices, marking a critical step toward the realization of all‐solution‐processed tandem solar cells.

## Conflicts of Interest

The authors declare that they have no conflicts of interest.

## Supporting information




**Supporting File**: advs73714‐sup‐0001‐SuppMat.docx.

## Data Availability

The data that support the findings of this study are available from the corresponding author upon reasonable request.
