# Supplementary material for: Overcoming Hole‐Extraction Barriers: A Facile PEDOT:PSS Interlayer Unlocks Record‐Low Voltage Deficit in PbS Quantum Dot Solar Cells
Source: Adv Sci (Weinh). 2026 Jan 9;13(16):e24275. doi: 10.1002/advs.202524275 (PMC13042711; doi:10.1002/advs.202524275)
Supplement: Supplementary file 1 — Supporting File: advs73714‐sup‐0001‐SuppMat.docx. [file ADVS-13-e24275-s001.docx]

**Supporting Information**

Overcoming Hole-Extraction Barriers: A Facile PEDOT:PSS Interlayer Unlocks Record-Low Voltage Deficit in PbS Quantum Dot Solar Cells

Shengkai Kang, Zixuan Meng, Yabing Wang, Kanwen Zheng, Sibo Huang, Di Zhang, Shitong Li, Cong Chen, Shenqing Ren, Yang Li, Chao Ding*, Dewei Zhao*

S. Kang, Z. Meng, Y. Wang, K. Zheng, S. Huang, D. Zhang, S. Li, Prof. C. Chen, Prof. S. Ren, Prof. Y. Li, Prof. C. Ding and Prof. D. Zhao

*College of Materials Science and Engineering & Institute of New Energy and Low-Carbon Technology, Engineering Research Center of Alternative Energy Materials & Devices of Ministry of Education, Sichuan University, Chengdu 610065, China*

*E-mail: [dc1107@scu.edu.cn](mailto:dc1107@scu.edu.cn) (Chao Ding)，d[ewei.zhao@scu.edu.cn](mailto:ewei.zhao@scu.edu.cn) and dewei_zhao@hotmail.com (Dewei Zhao)

**Keywords:** PbS Quantum Dot Solar Cells, Interface Engineering, PEDOT:PSS, Voltage Deficit

**Experimental Section**

**Materials**

Lead iodide (PbI_2_, 99+%), N,N′-Diphenylthiourea (DPhTA, 98%), lead oxide (PbO 98%), oleic acid (OA 90%), Oleylamine (OAm), 1-Octadecene (ODE, 90%), hexamethyldisilathiane (TMS-S, 98%), Zinc acetate dihydrate (ZnAc·2H_2_O, 98%), N,N’-dimethylformamide (DMF, 99.8% anhydrous), 3-Mercaptopropionic acid (MPA, 99%), Ethane-1,2-dithiol (EDT, 98%), Butylamine (BA, 99%), Toluene (TOL, 95%), Potassium hydroxide (KOH, >85%), Methanol (MeOH, 99%), Acetone (98%), Octane (98%), Chloroform (CF, 95%).

**Synthesis of PbS-OA CQDs**

The oleate-capped PbS (PbS-OA) CQDs were synthesized according to the literature with some modifications.^[1-2]^ A mixture of PbO (6 mmol), oleic acid (OA, 15 mmol), and 1-Octadecene (ODE, 50 ml) was evacuated under vacuum at 100 °C for 1 h. Heating was then ceased (while retaining the heating mantle), and the reaction mixture was allowed to cool gradually until the temperature reached 80 °C. At this point, a solution of trimethyldisilane (TMS, 2 mmol) in ODE (10 mL) was rapidly injected. The mixture was cooled to room temperature and purified as follows: nanoparticles were isolated by the addition of acetone followed by centrifugation. Further purification was performed via dispersion and precipitation cycles using toluene as the solvent and a mixture of acetone and methanol as anti-solvents. The final product was dispersed in n-octane at a concentration of 100 mg/mL.

**Synthesis and Purification of ZnO NCs**

ZnO nanoparticles were synthesized according to the literature with some modifications.^[3-5]^ In a typical synthesis process , 2.95 g of zinc acetate dihydrate (ZnAc·2H_2_O) was dissolved in 125 mL of methanol, and the temperature of the reaction bath was set to 63.5 °C under vigorous stirring. In a separate vial, 1.48 g of potassium hydroxide (KOH, >85%) was dissolved in 65 mL of methanol. The potassium hydroxide solution was then added dropwise into the zinc acetate solution under continuous stirring, while maintaining the reaction temperature at 63.5 °C. The reaction conditions were kept unchanged for 3 hours to facilitate the formation and growth of ZnO nanocrystals (NCs). After the reaction was completed, the heat source was removed, and the reaction bath was allowed to cool to room temperature. The solution was then transferred into centrifuge tubes and centrifuged at 3000 rpm for 2 minutes. Subsequently, the supernatant was discarded, and an equivalent volume of methanol was added to each centrifuge tube. The nanocrystals were dispersed in the methanol solution using a glass pipette, followed by another centrifugation step. This purification process was repeated three times before the final dispersion. Finally, the ZnO nanocrystals were dispersed in 20 mL of a 1:1 mixture of methanol and chloroform.

**Synthesis and Purification of PbS CQD Inks**

PbS CQDs were synthesized based on a previous report with some modifications.^[6-8]^ Lead iodide (PbI₂, 6 mmol), N,N′-Diphenylthiourea (DPhTA, 1 mmol), and 3-Mercaptopropionic acid (MPA , 16 μL) were dissolved in 9 mL of N,N’-dimethylformamide (DMF) and stirred at room temperature under a dry atmosphere. After complete dissolution, 1 mL of Butylamine (BA) solution was injected into the above PbI₂ solution, upon which the mixture immediately turned black. After 10 minutes, 25 mL of toluene was added as an anti-solvent, and the mixture was centrifuged at 8000 rpm for 5 minutes to isolate the colloidal quantum dot (CQD) solid. The final product was dispersed in DMF at a concentration of 1000 mg/mL.

**Device Fabrication**

ITO-coated glass substrates were sequentially cleaned with detergent, deionized water, and ethanol, each for 30 minutes, followed by ozone treatment for 15 minutes prior to use. For solar cell fabrication, a ZnO nanoparticle layer was spin-coated at 3000 rpm for 30 s. A PbS CQD ink in DMF was then spin-coated at 2000 rpm for 40 s under a dry atmosphere and annealed at 70 °C for 10 minutes. For the hole-transport layers (HTLs), a solution of 20 mg mL⁻¹ PbS-OA CQDs in n-octane was spin-coated at 2500 rpm for 15 s, followed by treatment with an EDT solution in acetonitrile (v:v = 1:1000) for 30 s and rinsing with acetonitrile. This spin-coating and ligand exchange procedure was repeated twice. Subsequently, a PEDOT:PSS solution diluted with methanol at a 1:2 volume ratio was spin-coated at 4000 rpm for 60 s and annealed at 70 °C for 10 minutes. Finally, a 100 nm-thick Ag electrode was thermally evaporated on the resulting stack. The resultant active area of 0.0985 cm^2^.

**Measurement and Characterization**

The current density–voltage (*J-V*) characteristics were measured using a Keysight B2901A source measure unit under simulated AM 1.5G illumination (100 mW cm⁻²) in a nitrogen-filled glovebox, with a scan rate of 50 mV s⁻¹ and a dwell time of 100 ms. The light intensity was calibrated using a certified silicon reference cell (SRC-00205, Enli Tech) coupled with a solar simulator (SS-F5-3A, Enli Tech). Devices with an active area of 0.0985 cm² were characterized through a black shadow mask featuring an aperture area of 0.0576 cm². External quantum efficiency (EQE) spectra were acquired by a computer-controlled quantum efficiency measurement system (QE-R, Enli Tech). Aging tests under oxygen atmosphere were conducted in a temperature-controlled desiccator (QHD260, ZISOdry) maintained at 20 °C with a relative humidity (RH) of < 20%. Electrochemical impedance spectroscopy (EIS), Mott-Schottky analysis, transient photovoltage (TPV), and transient photocurrent (TPC) measurements were performed on an electrochemical workstation (Zennium, Zahner, Germany).

Scanning electron microscopy (SEM) measurement was performed using a Zeiss Sigma 300 with an extra high tension of 10 kV. Atomic force microscopy (AFM) images were performed using a Bruker Dimension Icon in tapping mode. Kelvin probe force microscopy (KPFM) images were performed using Bruker Nano Inc DI Multi-Mode 8. UPS/XPS tests were made by spin-coating film (the same details as device fabrication) onto ITO for decay test. X-ray photoelectron spectroscopy (XPS) was performed on a photoelectron spectrometer (Thermo Scientific K-Alpha). Ultraviolet Photoelectron Spectroscopy (UPS) data were acquired on a Thermo Fisher Scientific ESCALAB XI+ system. UV-Vis NIR spectra were recorded on a Perkin Elmer model Lambda 950.

**Statistical Analysis**

Data analysis was performed using Origin 2021. Photovoltaic parameters (PCE, *V*_oc_, *J*_sc_, FF) are presented as mean ± standard deviation. The sample size for statistical distribution analysis was n=20 for each group. No specific data transformation or outlier removal was applied.

Figure S1. (a) Schematic illustration of the PbS quantum dot solar cell (CQDSC) structure. (b) Cross-sectional SEM image of the complete device.

Figure S2. Contact angle measurements of different precursor solutions on the hydrophobic PbS-EDT substrate: (a) pristine PEDOT:PSS (43.6°), (b) PEDOT:PSS-MeOH (11.3°), (c) PEDOT:PSS-OA (69.6°), and (d) the synergistic PEDOT:PSS-MeOH-OA (32.6°) solution.


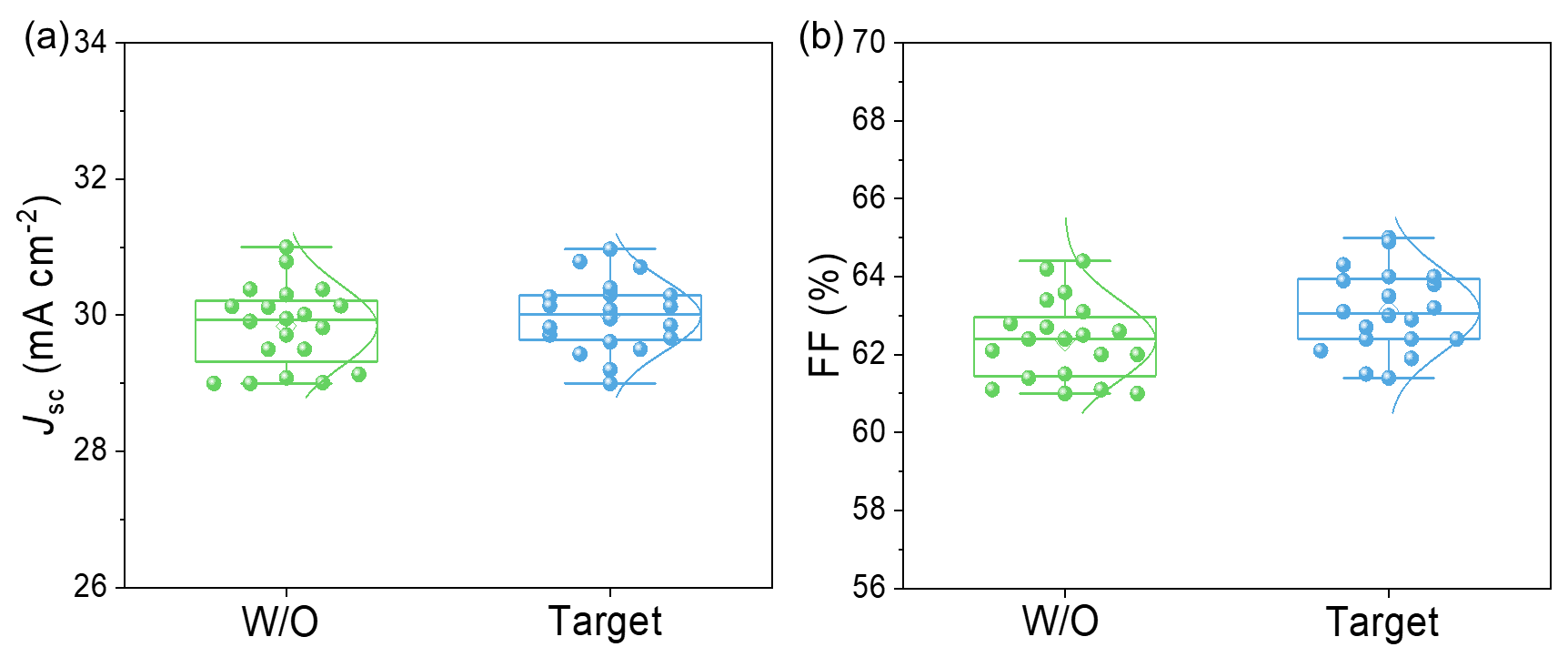


Figure S3. Statistical distribution of *J*_sc_ and FF for the W/O (control) and Target (optimized) devices, derived from 20 individual cells for each group.

Figure S4. EQE spectrum and corresponding integrated *J*_sc_ for the W/O (control) device, yielding an integrated *J*_sc_ of 28.21 mA/cm².

Figure S5. Fourier-transform infrared spectroscopy (FTIR) transmittance spectra for the pristine PEDOT:PSS, PEDOT:PSS-MeOH (W/O), and PEDOT:PSS-MeOH-OA (Target) films.

Figure S6. Photographs of the pH measurements for the precursor solutions: (a) pristine PEDOT:PSS, (b) PEDOT:PSS-MeOH (W/O), and (c) PEDOT:PSS-MeOH-OA (Target).

Figure S7. UV-Vis absorption spectra for the pristine PEDOT:PSS, PEDOT:PSS-MeOH (W/O), and PEDOT:PSS-MeOH-OA (Target) films.

Figure S8. Ultraviolet photoelectron spectroscopy (UPS) data for the key functional layers. (Right row) Secondary electron cutoff (SECO) spectra used to determine the work function (WF) values. (Left row) Valence band spectra used to determine the valence band maximum (VBM).


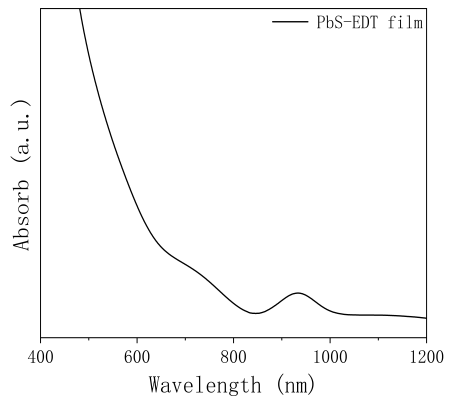

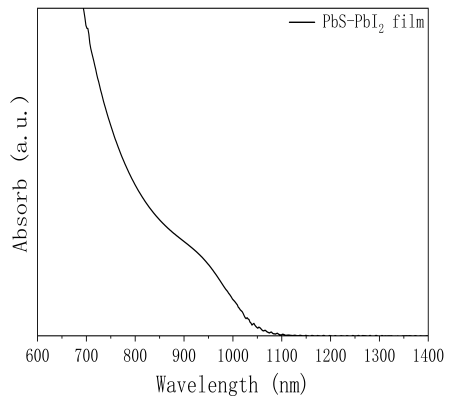

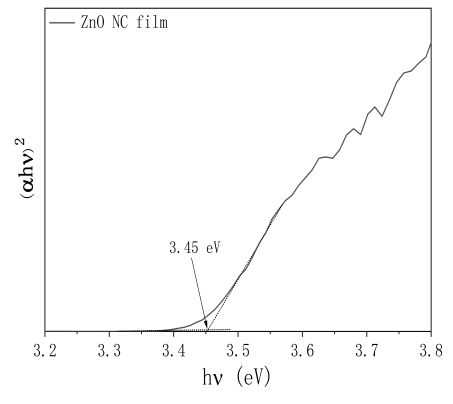


Figure S9. UV-Vis absorption spectra for (a) the PbS-PbI₂ absorber film and (b) the PbS-EDT HTL film, (c) Tauc plots derived from UV-Vis absorption spectra for ZnO NC film used to determine their respective optical bandgaps (*E*_g_).


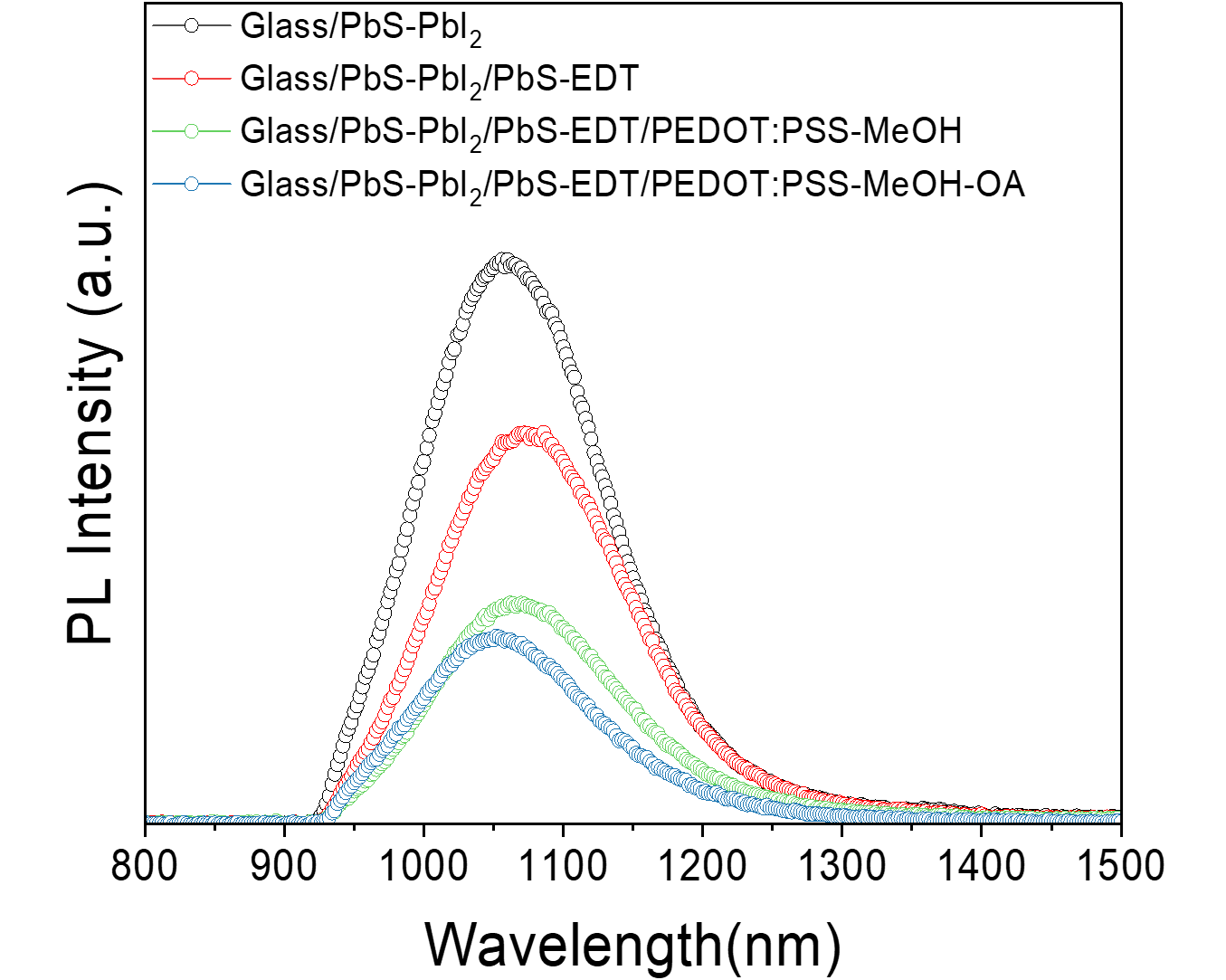


Figure S10. Steady-state PL spectra of the PbS-PbI₂ films with different hole transport layer configurations, corresponding to the samples analyzed in Figure 3d. The spectra correspond to Glass/PbS-PbI₂ (black), Glass/PbS-PbI₂/PbS-EDT (red), Glass/PbS-PbI₂/PbS-EDT/PEDOT:PSS-MeOH (green), and Glass/PbS-PbI₂/PbS-EDT/PEDOT:PSS-MeOH-OA (blue).

Figure S11. Equivalent circuit model used for fitting the Electrochemical Impedance Spectroscopy (EIS) data presented in Figure 3d. (*R*_s_: series resistance; *R*_ct_: charge transfer resistance; *R*_rec_: recombination resistance; *C*_geo_: geometric capacitance; *C*_μ_: chemical capacitance)

Table S1. Summary on bandgap, *V*_oc_ and *V*_oc_ loss for the PbS CQD solar cells with PCE >10%.

| Bandgap  (EQE_onset_) | *V*_oc_  (V) | *V*_oc_ loss  (V) | PCE  (%) | Reference |
| --- | --- | --- | --- | --- |
| 1.13 | 0.59 | 0.54 | 11.3 | ACS Nano. 2020, 14, 384 |
| 1.13 | 0.61 | 0.52 | 10.6 | Nano Lett. 2016, 16, 4630 |
| 1.15 | 0.63 | 0.52 | 11.2 | J. Mater. Chem. A 2020, 8, 4844 |
| 1.13 | 0.61 | 0.52 | 11.2 | J. Mater. Chem. A 2019, 7, 15951 |
| 1.21 | 0.69 | 0.52 | 11.7 | Small. 2022, 18, e2201387 |
| 1.11 | 0.6 | 0.51 | 11.2 | ACS Energy Lett. 2019, 4, 2850 |
| 1.18 | 0.67 | 0.51 | 14.28 | Adv. Mater. 2025, 2500562 |
| 1.18 | 0.67 | 0.51 | 13.89 | Adv. Mater. 2025, e12933 |
| 1.12 | 0.62 | 0.5 | 11.5 | Adv. Energy Mater. 2020, 10, 1902933 |
| 1.12 | 0.62 | 0.5 | 10.6 | Adv. Mater. 2018, 30, e1704871 |
| 1.13 | 0.64 | 0.49 | 10.2 | Adv. Mater. 2016, 28, 299 |
| 1.13 | 0.64 | 0.49 | 11.1 | ACS Appl. Energy Mater. 2020, 3, 5135 |
| 1.1 | 0.61 | 0.49 | 11.3 | Nat. Mater. 2017, 16, 258 |
| 1.13 | 0.64 | 0.49 | 11.5 | Adv. Funct. Mater. 2020, 30, 2000594 |
| 1.08 | 0.6 | 0.48 | 11.2 | Adv. Mater. 2017, 29, 1700749 |
| 1.13 | 0.65 | 0.48 | 11.4 | Adv. Energy Mater. 2019, 9, 1902809 |
| 1.13 | 0.65 | 0.48 | 13.4 | Adv. Sci. 2024, 2402756 |
| 1.09 | 0.62 | 0.47 | 12.4 | ACS Energy Lett. 2020, 5, 3224 |
| 1.14 | 0.66 | 0.47 | 13.6 | Chem. Commun. 2025, 61, 7426 |
| 1.11 | 0.65 | 0.46 | 12 | ACS Energy Lett. 2020, 5, 2335 |
| 1.08 | 0.63 | 0.45 | 12.3 | Adv. Mater. 2018, 30, e1803830 |
| 1.09 | 0.64 | 0.45 | 12.6 | Nature. 2019, 570, 96 |
| 1.09 | 0.64 | 0.45 | 13 | Adv. Mater. 2020, 32, e1906199 |
| 1.18 | 0.68 | 0.45 | 13.5 | Adv. Mater. 2023, 35, 2212184 |
| 1.08 | 0.64 | 0.44 | 13.2 | Adv. Energy Mater. 2020, 10, 2002084 |
| 1.1 | 0.66 | 0.44 | 13 | ACS Energy Lett. 2020, 5, 3452 |
| 1.14 | 0.7 | 0.44 | 12.3 | J. Mater. Chem. A 11(32), 17282 |
| 1.14 | 0.7 | 0.44 | 14.15 | [Nat. Energy](https://www.nature.com/nenergy). 2025,10,591 |
| 1.07 | 0.64 | 0.43 | 12 | Nat. Nanotechnol. 2018, 13, 456 |
| 1.08 | 0.65 | 0.43 | 13.3 | Nat. Commun. 2020, 11, 103 |
| 1.08 | 0.65 | 0.43 | 13.8 | Joule. 2020, 4, 1542 |
| 1.08 | 0.66 | 0.42 | 14 | Adv. Mater. 2020, 32, e2004985 |
| 1.08 | 0.66 | 0.42 | 15.4 | Adv. Energy Mater. 2022, 12, 2201676 |
| 1.08 | 0.67 | 0.41 | 12.1 | Nano Energy. 2019, 63, 103876 |
| 1.08 | 0.68 | 0.4 | 11.6 | Adv. Mater. 2018, 30, e1707572 |
| 1.08 | 0.7 | 0.38 | 10.9 | Adv. Mater. 2017, 29, 1703627 |
| 1.03 | 0.66 | 0.37 | 13.1 | Nat. Energy. 2019, 4, 969 |
| 1.06 | 0.71 | 0.35 | 12.8 | Adv. Mater. 2023, 35, 2207293 |
| **1.08** | **0.74** | **0.34** | **13.97** | **This work** |

Table S2. The summary of bandgap, *V*_oc_ and *V*_oc_ loss for other state-of-the-art photovoltaic technologies.

| Name | Bandgap  (EQE onset) | *V*_oc_  (V) | *V*_oc_ loss  (V) | PCE  (%) | Reference |
| --- | --- | --- | --- | --- | --- |
| PVK | 1.53 | 1.229 | 0.301 | 25.22 | Nat. Commun. 2025, 16, 190 |
| Si | 1.08 | 0.7449 | 0.3351 | 27.8 | Prog Photovolt Res Appl. 2025, 33, 795-810 |
| GaAs | 1.4 | 0.998 | 0.402 | 29.1 | Prog Photovolt Res Appl. 2025, 33, 795-810 |
| InP | 1.34 | 0.939 | 0.401 | 24.2 | US Patent 9,590,131 B2,2017. |
| CdTe | 1.45 | 0.8759 | 0.5741 | 21 | Prog Photovolt Res Appl. 2025, 33, 795-810 |
| CZTSSe | 1.03 | 0.53 | 0.5 | 14.13 | Nat Energy 2023, 8, 526 |
| Organic | 1.43 | 0.923 | 0.507 | 21 | Nat. Mater. 2025, 24, 1626–1634 |
| PVK | 1.265 | 0.911 | 0.354 | 24.9 | Nature. 2025, 648, 600-606 |

Table S3. Summary of parameters obtained from the deconvolution of high-resolution S 2p XPS spectra (shown in Figure 2a) for the pristine PEDOT:PSS, PEDOT:PSS-MeOH (W/O), and PEDOT:PSS-MeOH-OA (Target) films, detailing the binding energies and relative area ratios of the fitted PEDOT and PSS components.

| Composition  Sample | PEDOT | | | | PSS | | | |
| --- | --- | --- | --- | --- | --- | --- | --- | --- |
|  | 2P_1/2_ | 2P_3/2_ | 2P_1/2_ | 2P_3/2_ | 2P_1/2_ | 2P_3/2_ | 2P_1/2_ | 2P_3/2_ |
|  | position | area | position | area | position | area | position | area |
| PEDOT:PSS | 163.7ev | 0.13 | 165.08 | 0.16 | 168.06ev | 1 | 169.25ev | 0.66 |
| PEDOT:PSS -MeOH | 163.8ev | 0.13 | 164.99 | 0.16 | 168.06ev | 1 | 169.25ev | 0.66 |
| PEDOT:PSS -MeOH-OA | 163.8ev | 0.13 | 164.88 | 0.13 | 168.18ev | 1 | 169.37ev | 0.62 |

Table S4. Summary of Hall effect measurement data for the pristine PEDOT:PSS, PEDOT:PSS-MeOH (W/O), and PEDOT:PSS-MeOH-OA (Target) films, detailing the measured resistivity, mobility, carrier concentration, Hall coefficient, and f-factor.

| Sample | Resistivity  (Ω·cm) | Mobility (cm²/V·s) | Carrier Concentration  (cm⁻³) | Hall Coefficient (cm³/C) | f-factor |
| --- | --- | --- | --- | --- | --- |
| PEDOT:PSS | 1.64×10^2^ | 1.15 | 3.32×10^16^ | −1.88×10^2^ | 0.977 |
| PEDOT:PSS-MeOH | 1.28×10^2^ | 1.28 | 3.79×10^16^ | −1.65×10^2^ | 0.998 |
| PEDOT:PSS-MeOH-OA | 1.14×10^2^ | 1.39 | 3.94×10^16^ | −1.58×10^2^ | 0.935 |

**References**

[1] C. Ding, D. Wang, D. Liu, H. Li, Y. Li, S. Hayase, T. Sogabe, T. Masuda, Y. Zhou, Y. Yao, Z. Zou, R. Wang, Q. Shen, *Adv. Energy Mater.* **2022**, *12* (35), 2201676.

[2] Y. Wang, K. Lu, L. Han, Z. Liu, G. Shi, H. Fang, S. Chen, T. Wu, F. Yang, M. Gu, S. Zhou, X. Ling, X. Tang, J. Zheng, M. A. Loi, W. Ma, *Adv. Mater.* **2018**, *30* (16), 1704871.

[3] B. Zhang, N. Xu, J. Wan, H. Li, J. Xia, J. Zhu, J. Xu, H. Chen, W. Chen, G. Zeng, Y. Li, Y. Li, *Energy Environ. Sci.* **2025**, *18* (21), 9550.

[4] S. Fang, J. Huang, R. Tao, Q. Wei, X. Ding, S. Yajima, Z. Chen, W. Zhu, C. Liu, Y. Li, N. Yin, L. Song, Y. Liu, G. Shi, H. Wu, Y. Gao, X. Wen, Q. Chen, Q. Shen, Y. Li, Z. Liu, Y. Li, W. Ma, *Adv. Mater.* **2023**, *35* (21), 2212184.

[5] M. A. Hines, G. D. Scholes, *Adv. Mater.* **2003**, *15* (21), 1844.

[6] Y. Wang, Z. Liu, N. Huo, F. Li, M. Gu, X. Ling, Y. Zhang, K. Lu, L. Han, H. Fang, A. G. Shulga, Y. Xue, S. Zhou, F. Yang, X. Tang, J. Zheng, M. Antonietta Loi, G. Konstantatos, W. Ma, *Nat. Commun.* **2019**, *10* (1), 5136.

[7] Y. Liu, H. Wu, G. Shi, Y. Li, Y. Gao, S. Fang, H. Tang, W. Chen, T. Ma, I. Khan, K. Wang, C. Wang, X. Li, Q. Shen, Z. Liu, W. Ma, *Adv. Mater.* **2023**, *35* (5), 2207293.

[8] J. Liu, J. Qiao, K. Zhou, J. Wang, R. Gui, K. Xian, M. Gao, H. Yin, X. Hao, Z. Zhou, L. Ye, *Small* **2022**, *18* (19), 2201387.
